# Use of intrathoracic pressure regulation therapy in breathing patients for the treatment of hypotension secondary to trauma

**DOI:** 10.1186/s13049-017-0450-5

**Published:** 2017-10-30

**Authors:** Victor A. Convertino, Brent A. Parquette, David A. Wampler, Craig A. Manifold, David A. Lindstrom, Lori L. Boland, Nathan T. Burkhart, Keith G. Lurie, Charles J. Lick

**Affiliations:** 1 Battlefield Health & Trauma Center for Human Integrative Physiology US Army Institute of Surgical Research, 3698 Chambers Pass, Fort Sam Houston, Houston, TX 78234 USA; 2 Lucas County EMS, 2144 Monroe Street, Toledo, OH 43604 USA; 3 University of Texas Health Science Center, 7703 Floyd Curl Drive, San Antonio, TX 78229 USA; 4 Allina Health Emergency Medical Services, 167 Grand Avenue, St. Paul, MN 55102 USA; 5 ZOLL Medical, 1905 County Road C West, Roseville, MN 55113 USA; 6 University of Minnesota, 420 Delaware Street SE, Minneapolis, MN 55455 USA

**Keywords:** Hypotension, Trauma, Impedance threshold device, Hemodynamics, Hypotensive resuscitation

## Abstract

**Background:**

Intrathoracic pressure regulation (IPR) therapy has been shown to increase blood pressure in hypotensive patients. The potential value of this therapy in patients with hypotension secondary to trauma with bleeding is not well understood. We hypothesized that IPR would non-invasively and safely enhance blood pressure in spontaneously breathing patients with trauma-induced hypotension.

**Methods:**

This prospective observational cohort study assessed vital signs from hypotensive patients with a systolic blood pressure (SBP) ≤90 mmHg secondary to trauma treated with IPR (ResQGARD™, ZOLL Medical) by pre-hospital emergency medical personnel in three large US metropolitan areas. Upon determination of hypotension, facemask-based IPR was initiated as long as bleeding was controlled. Vital signs were recorded before, during, and after IPR. An increased SBP with IPR use was the primary study endpoint. Device tolerance and ease of use were also reported.

**Results:**

A total of 54 patients with hypotension secondary to trauma were treated from 2009 to 2016. The mean ± SD SBP increased from 80.9 ± 12.2 mmHg to 106.6 ± 19.2 mmHg with IPR (*p* < 0.001) and mean arterial pressures (MAP) increased from 62.2 ± 10.5 mmHg to 81.9 ± 16.6 mmHg (*p* < 0.001). There were no significant changes in mean heart rate or oxygen saturation. Approximately 75% of patients reported moderate to easy tolerance of the device. There were no safety concerns or reported adverse events.

**Conclusions:**

These findings support the use of IPR to treat trauma-induced hypotension as long as bleeding has been controlled.

## Background

Hypotension in the battlefield and civilian setting remains a leading cause of potentially reversible morbidity and mortality. It is estimated that death from hemorrhage in the combat setting could be reduced by up to 30% if a patient’s mean arterial pressure (MAP) can be maintained until definitive therapy is available. [1] A target for MAP has not been clearly identified by controlled randomized studies, but based on current evidence guidelines recommend achieving a MAP of 65 mmHg or more. [2] Current therapies designed to manage hypotension include hemorrhage control with tourniquets and administration of saline, blood substitutes, dried plasma, and blood products. Unfortunately, intravenous access and availability of such treatments are often limited by the clinical setting, the location of the wounded patient, and logistical considerations.

Based upon lessons learned from the battlefield, modern military and civilian treatment recommendations stress the need to provide hypotensive resuscitation with a target systolic blood pressure (SBP) of 80 to 90 mmHg in order to minimize interference with normal hemostatic response and to reduce the risk of over-resuscitation. [3] With the need for extended care, especially the anticipated military and civilian need for ‘prolonged field care’ [4, 5] that may extend hours past the exhaustion of all available conventional life-saving interventions in the field, the clinical provider will be required to initiate goal-directed resuscitation with the objective of “delivering a casualty to a definitive care facility in the best condition possible.” [6] As such, alternative approaches are needed to safely buy time and preserve a patient’s condition until definitive care can be administered, especially in remote regions where it can take many hours until that additional care is available.

In 2006, the application of a novel concept called intrathoracic pressure regulation (IPR) therapy, using an inspiratory impedance threshold device (ITD), was first described to increase venous return, cardiac output, and ultimately survival rates in animal models of hemorrhagic shock. [7, 8] Subsequently, this technology was evaluated in human volunteers and similarly improved cardiac output and blood pressure to levels that were associated with increased tolerance to central hypovolemia and hypotension. [9–11] These evaluations demonstrated that a simple, small, light-weight non-invasive device could be used to harness the work of breathing to increase and sustain MAP. [12, 13] Use of IPR with an inspiratory resistance of 7 cm H_2_O, created by breathing through an ITD, does not cause a secondary hypertensive response. Breathing through the ITD is associated with an increased work of breathing equivalent to that required to take a walk, it is well tolerated by most people with normal respiratory function [14] and does not significantly impede exhalation. Investigations conducted at NASA and the Defense Department laboratories also showed that use of IPR can mitigate the onset of cerebral hypoperfusion during the treatment of hypotension by modulating brain blood flow, oscillations in blood flow, and autonomic function through enhanced cardiac output and lower intracranial pressure [15, 16].

Building on the studies in normal volunteers and animal experiments that showed IPR could buy time and extend the “golden hour” in the setting of life-threatening blood loss, IPR studies were conducted in hypotensive patients in emergency departments, blood banks, renal dialysis units, syncope clinics, and by emergency medical service (EMS) responders to treat hypotension from a wide variety of causes. [10, 17–21] These studies demonstrated that use of IPR improved MAP in patients with clinical hypotension, defined as a pre-treatment SBP of <90 mmHg, with and without concurrent administration of intravenous fluids or vasopressors.

To date, there have been limited reports on the use of IPR for the treatment of hypotension secondary to trauma. Demonstration that IPR can be used to safely and effectively treat trauma, after bleeding has been controlled, is needed before such a device could be deployed and widely used, particularly in combat casualties who have suffered severe blood loss. The purpose of the current observational study was to prospectively evaluate whether IPR can be used to safely and effectively treat hypotension secondary to traumatic injury in spontaneously breathing patients without known ongoing hemorrhage. Hemodynamic data and survival to hospital discharge were collected along with an assessment of patient comfort to assess IPR safety and efficacy in the treatment of hypotension secondary to all-cause trauma.

## Methods

### Setting

Anonymous quality assurance data from events that occurred between 2009 and 2016 were collected by EMS personnel in three study sites: San Antonio, Texas (San Antonio Fire Department); Lucas County, Ohio (Lucas County EMS); and Minneapolis/St. Paul and outstate Minnesota (Allina Health EMS). These data were collected by each of the sites during their initial evaluation of the IPR device and were combined for this manuscript; this was an evaluation of outcome data from three EMS systems using the IPR device. The three sites represent a cross-section of large population EMS services and include a fire-based EMS service in a large metro area (San Antonio), a county-wide ALS/paramedic service serving urban and suburban areas (Lucas County), and a widely distributed ALS/BLS service covering both urban and rural communities (Minnesota). Permissions to gather, analyze, and report these data were provided by respective local Institutional Review Boards (IRBs) in compliance with their individual quality assurance and improvement regulations.

### IPR use

**Fig. 1:**
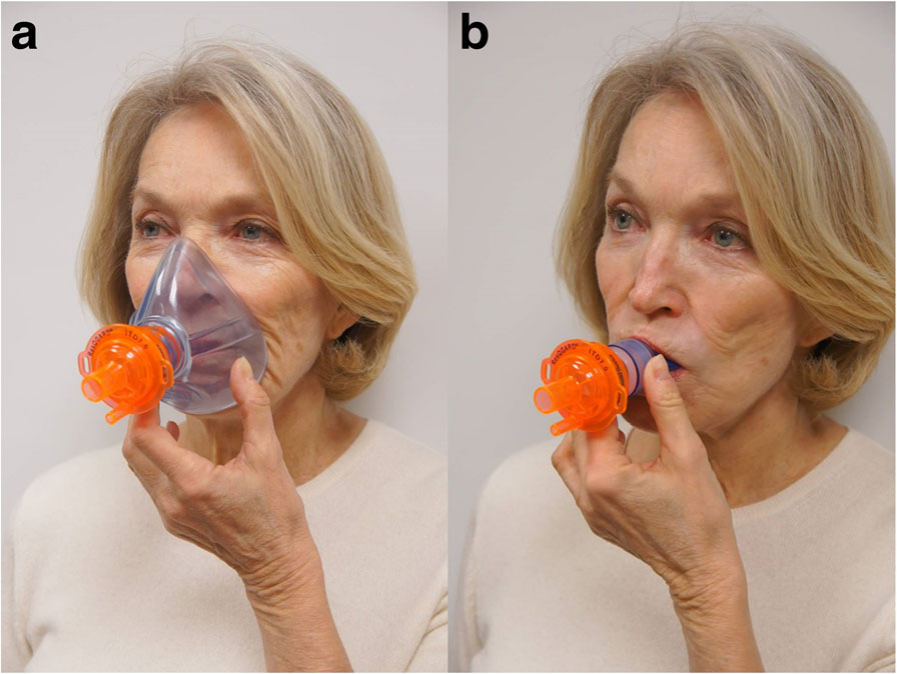
ITD attached to **a** facemask and **b** mouthpiece

IPR was delivered via the use of a commercially available ITD (ResQGARD^®^, ZOLL Medical; Roseville, MN) that is US FDA approved to increase circulation in patients with low blood circulation. Use of IPR by emergency medical service (EMS) personnel has been previously described by investigators from both San Antonio, TX, and Lucas County, OH. [18, 19] Routine use of the ITD is included in the hypotension treatment protocols at all three sites involved in this study and was thus uniformly available for use by either Basic (BLS) or Advanced Life Support (ALS) personnel on scene. Once it was determined that a patient with hypotension was spontaneously breathing and met IPR use criteria (exclusion criteria included complaints of chest pain or shortness of breath, pulmonary hypertension, active congestive heart failure, known aortic stenosis, inability to maintain airway, pulmonary edema, or dilated cardiomyopathy), the device was attached to either a facemask or a mouthpiece (Fig. 1) and the patient was instructed to take slow deep breaths through the ITD. It sometimes took one to two minutes of coaching by EMS personnel before the patient felt comfortable breathing through the device.

EMS personnel were allowed and instructed to provide concomitant standard therapies for hypotension, including reversal of other potential causes of hypotension such as correcting hyperthermia and administration of fluids, oxygen, vasopressors, and/or patient positioning as appropriate and directed by standing protocols. Use of IPR did not interfere with or preclude standard therapies and overall patient care. Physiological parameters were assessed before, during, and after IPR as part of the standard clinical protocol. Similarly, administration of standard therapies such as intravenous fluid administration and oxygen (O_2_) were recorded and monitored.

### Data collection and statistical analysis

The treating first responder or paramedic assessed outcome data by non-invasively measuring SBP and diastolic blood pressure (DBP), heart rate (HR), and O_2_ saturation (SpO_2_), and then used a questionnaire to assess patient comfort during therapy administration (as reported by the patient) and patient tolerance of the device (as reported by EMS personnel). Data collection was performed by abstraction from medical records at each site. Patients with hypotension secondary to trauma were included in these analyses.

The primary study endpoint was SBP; pre-IPR values were compared with SBP during IPR use. Secondary endpoints included HR, SpO_2_, respiratory rate (RR), and adverse events. Values are reported as mean ± SD. A paired Student’s T-test was used to determine the probability that any differences between endpoints did not exist by greater than chance (i.e., *p* < 0.05).

## Results

Data were obtained from 54 patients with hypotension secondary to trauma and analyzed and described in these results. The average patient age was 54.6 ± 18.8 years, and 31 patients were male. Although the etiology of the trauma varied, six of the fifteen causes of trauma shown in Table 1 accounted for at least 33% (18/54) of the patients’ sustained injuries that were associated with hemorrhage.

**Table 1:** Chief Complaint of Trauma Patients Treated with IPR

Chief Complaint	All Patients (*n* = 54)	
	n	%
Syncope	9	17
Fall	8	15
Motor vehicle collision	7	13
Gunshot wound^a^	4	7
Laceration^a^	4	7
GI bleed^a^	3	6
Hemorrhage^a^	3	6
Hypotension	3	6
Non-specified	3	6
Stabbing^a^	3	6
Altered consciousness	2	4
Attempted suicide	2	4
Fight^a^	1	2
Homicide	1	2
Seizure	1	2

^a^Trauma associated with hemorrhage

SBP before and during IPR are shown in Table 2. SBP increased in 49/54 (90.7%) patients treated with IPR. In the five patients not exhibiting an increase in SBP, the average systolic blood pressure before IPR was 96.4 ± 11.7 mmHg and during IPR was 95.2 ± 12.0 mmHg.

**Table 2:** Hemodynamic and Respiratory Values for Trauma Patients Treated with IPR

	Patients with before and during data	Average before	Average during	Average change
SBP	54	80.9 ± 12.2	106.6 ± 19.2	25.7 ± 21.13
DBP	53	52.9 ± 10.8	69.4 ± 17.0	16.5 ± 18.4
MAP	53	62.2 ± 10.5	81.9 ± 16.6	19.7 ± 18.3
HR	51	83.4 ± 17.6	83.2 ± 17.5	−0.1 ± 11.6
RR	47	17.6 ± 3.5	18.0 ± 4.1	0.4 ± 3.5
SaO2	39	96.6 ± 3.7	97.9 ± 4.1	1.3 ± 3.8

**Fig. 2:**
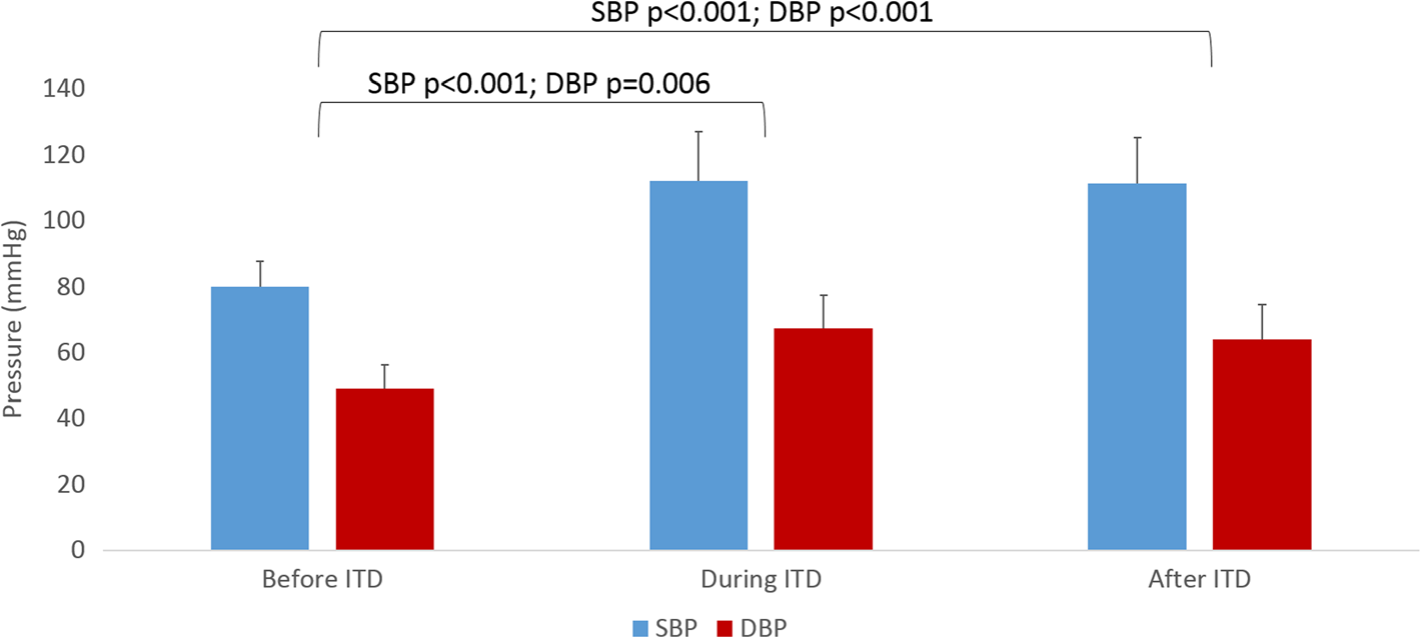
Average SBP and DBP in Patients with Data Before, During, and After IPR Use (*n* = 10)

Overall, the mean SBP increased from 80.9 ± 12.2 mmHg to 106.6 ± 19.2 mmHg with IPR (*p* < 0.001). Overall MAP increased with IPR from 62.2 ± 10.5 mmHg to 81.9 ± 16.6 mmHg (*p* < 0.001). IPR did not significantly alter heart rate, respiratory rate, or O_2_ saturation. All patients survived to hospital discharge. One site collected hemodynamic and respiratory values upon IPR discontinuation or arrival to hospital and transfer of patient care to emergency department staff. In the ten patients with data reported after IPR was discontinued, the average SBP was 111.2 ± 13.8 mmHg, essentially unchanged from a mean during IPR level of 111.9 ± 15.1 mmHg. (Fig. 2).

**Fig. 3:**
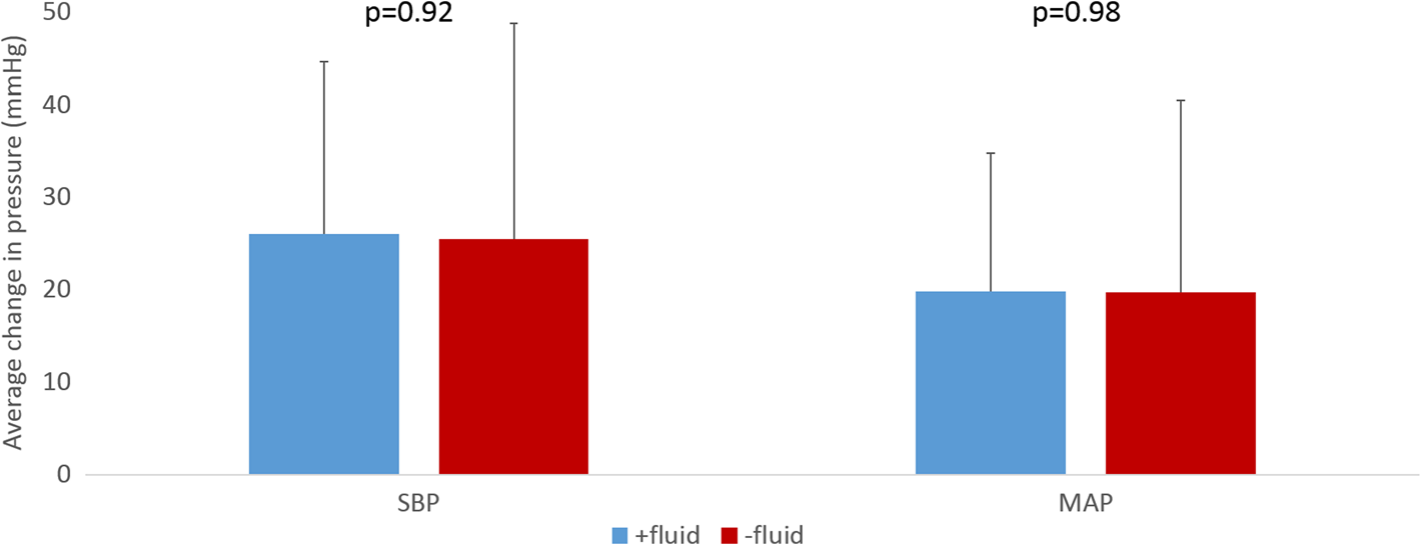
Change in SBP and MAP by Fluid Administration

Fluids were administered concurrently with IPR in 24/54 patients. As shown in Fig. 3, there was no difference in the change in SBP or MAP with concurrent fluid administration.

**Fig. 4:**
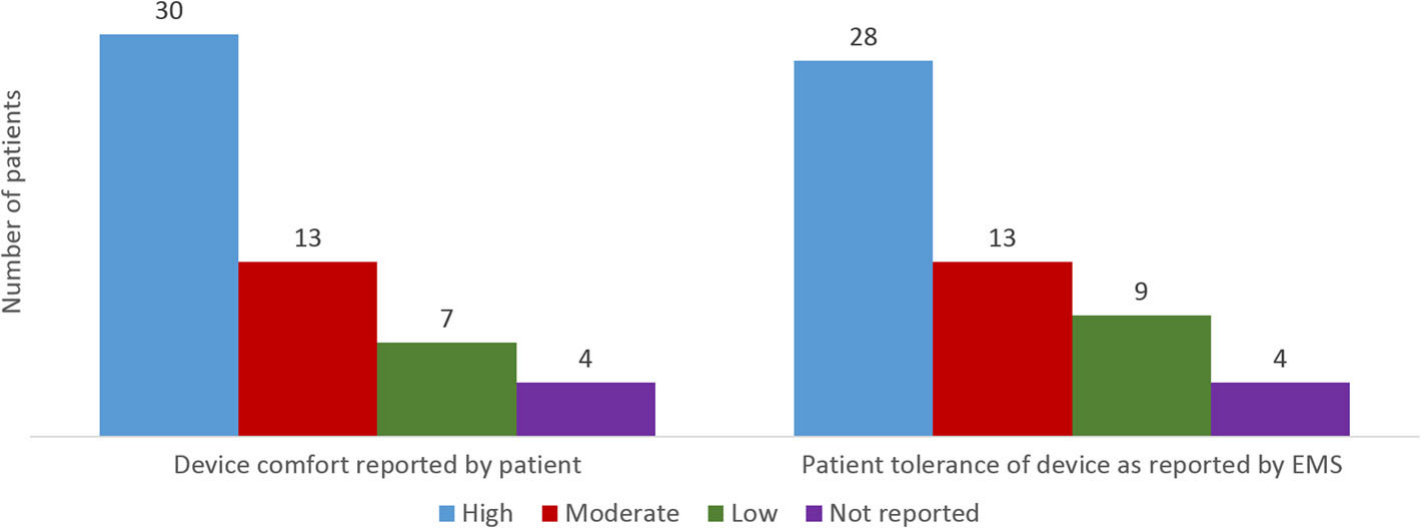
Device Comfort and Patient Tolerance of Device

An assessment of patient comfort revealed that most were able to successfully and comfortably breathe through the ITD (Fig. 4). The majority of patients reported that breathing through the ITD was comfortable and only 7/50 patients (14%) reported it was difficult (4 patients did not report). Additionally, EMS personnel reported 82% (41/50) of patients were able to tolerate the device well whereas 18% (9/50) of patients reported it was more difficult to breathe through the ITD but still used it for some period of time. Hemodynamic values were recorded for those 9 patients while the ITD was being used and before they removed it.

## Discussion

These results in patients with hypotension secondary to trauma in the current study were consistent with findings from prior preclinical and clinical studies that showed that resistance breathing could be harnessed to improve circulation and treat hypotension. The severity of trauma in the present study was sufficient to cause symptoms and resulted in a consistent SBP of <90 mmHg prior to treatment. In all cases, the patients were able to breathe spontaneously. On average, application of IPR therapy increased SBP by ~26 mmHg and provided symptomatic improvement. There were five patients in whom SBP did not increase with use of IPR, though four of the five patients presented with SBP > 90 mmHg and it could be argued that they were not primary candidates to receive the therapy. For the remaining case, we speculate there may not have been enough sympathetic tone or autonomic reserve to help compensate for the blood loss: thus even if central blood volume is increased and cardiac preload is enhanced, the blood pressure remains low due to reduced sympathetic tone. Another potential reason for the non-responders is the inability to generate a negative pressure while breathing on the IPR device. It was previously reported in one study that the single non-responder in that randomized blinded study with normal volunteers failed to generate a significant negative pressure upon inspiration through the IPR device when compared with other subjects. [9] These results underscore the importance of breathing mechanics in providing a positive ITD effect through generation of a minimum level of negative pressure to optimize the patient-powered thoracic pump mechanism that underlies the function of the ITD. Most patients tolerated use of the IPR device and heart rate, O_2_ saturation, and respiratory rate remained constant with application of the therapy. In addition, SBP and DBP were maintained even after removal of the IPR device in patients where before, during, and after values were reported. There were no safety concerns raised by the current study. Importantly, IPR therapy was not used in patients with known or suspected ongoing bleeding.

One of the important findings from this study is that increases in SBP and MAP observed with IPR were similar in magnitude to those achieved with IPR plus fluid administration and that co-administration of intravenous saline and IPR did not additionally increase SBP or MAP when compared with IPR alone. These findings, consistent with prior studies, suggest that IPR could be safely used as a first line therapy, especially in the absence of intravenous access or exhaustion of fluids in a prolonged field care situation. The findings are consistent with the known physiological benefit: IPR helps to maintain stroke volume and cardiac output in the setting of a significant decrease in circulating blood volume. The ability of IPR to maintain circulating blood volume without the need for intravenous saline suggests that it has the potential to play an important therapeutic role in hypotensive patients in the battlefield and in civilian settings once hemorrhage is controlled. The authors have been unable to find prospective, randomized trials that focused specifically on prehospital fluid resuscitation for trauma patients in shock from controlled hemorrhage. However, there have been animal models that address this question. [22, 23] Similarly, these prospective randomized animal studies have shown a definitive improvement in survival with use of IPR in the setting of a controlled hemorrhage. Advantages of using IPR by itself, at least as the first line of therapy, include lack of dilution of endogenous clotting factors and reduction in the risk of an overshoot in the systolic blood pressure that can ‘pop the clot’.

Use of IPR in the treatment of patients with trauma and hypotension in the setting of controlled bleeding provides a clinical benefit that is consistent with the current goal of providing permissive hypotension. It can restore some measure of perfusion without increasing the blood pressure sufficiently to disrupt clot formation or create dilutional coagulopathy. This notion is supported by the results of the present study in which there was no report of increased bleeding with IPR in the trauma cases caused by injuries associated with hemorrhage. Moreover, IPR can be used to obtain many of the clinical objectives of fluid resuscitation for casualties in hemorrhagic shock but without administration of fluids, including to 1) assist in the body’s ability to form clots at sites of active bleeding, 2) minimize adverse effects (edema and dilution of clotting factors) resulting from iatrogenic resuscitation injury, 3) help restore adequate central intravascular volume and organ perfusion prior to definitive surgical hemorrhage control, and 4) optimize oxygen carrying capacity.

This observational study has limitations including the fact that it was not prospective, blinded or randomized by design and there was no separate control group – each subject served as their own control with “before” measurements used as baseline data. Additionally, total duration of IPR provided for each patient was not available. Earlier randomized human studies in non-traumatic hypotension have shown a similar benefit to what was observed in this study, [24] but in the setting of hypotension secondary to trauma it is often difficult to obtain consent prior to device use. Moreover, in these patients, time is of the essence and immediate intervention is needed to prevent further hemodynamic deterioration and harm. In addition, the decision to co-administer fluids or vasopressors was made by the first responder or paramedic as a standard of care, but added variability to the statistics. Fluids may not have been available for some patients and use of concomitant fluids and vasopressors was not controlled or standardized across sites; interventions and care were to be administered according to each agency’s standard procedures. In light of this variability, the statistically significant elevation in the primary outcome variable (i.e., blood pressure) with a sample size of only 54 patients provides a compelling argument to support the notion of a significant intervention effect of IPR. Furthermore, the results from this study and prior EMS studies that included application of IPR suggest that the same hemodynamic benefit can be achieved with or without fluids.

## Conclusions

While additional studies of IPR in patients with trauma are needed to further define the potential and limits of resistance breathing for the treatment of hypotension, the current study, when combined with data from prior studies in hundreds of patients and test subjects and multiple animal models of shock, support the use of IPR for the safe and effective treatment of symptomatic hypotension in the traumatic patient population as long as bleeding has been controlled and the clinical suspicion of internal bleeding is low.

## Abbreviations

DBP: Diastolic blood pressure
EMS: Emergency medical service
HR: Heart rate
IPR: Intrathoracic pressure regulation
IRB: Institutional review board
ITD: Impedance threshold device
MAP: Mean arterial pressure
RR: Respiratory rate
SBP: Systolic blood pressure
